# Effects of dietary macronutrient composition and feeding frequency on fasting and postprandial hormone response in domestic cats

**DOI:** 10.1017/jns.2013.32

**Published:** 2013-12-03

**Authors:** Ping Deng, Tonya K. Ridge, Thomas K. Graves, Julie K. Spears, Kelly S. Swanson

**Affiliations:** 1 Department of Animal Sciences, University of Illinois, Urbana, IL, USA; 2 Department of Veterinary Clinical Medicine, University of Illinois, Urbana, IL, USA; 3 Nestlé Purina PetCare, St Louis, MO, USA; 4 Division of Nutritional Sciences, University of Illinois, Urbana, IL, USA

**Keywords:** Dietary macronutrients, Feline nutrition, Leptin, Ghrelin, BW, body weight, DAUC, decremental AUC, DXA, dual-energy X-ray absorptiometry, HC, high carbohydrate, HF, high fat, HP, high protein, IAUC, incremental AUC, ME, metabolisable energy, NAUC, net AUC

## Abstract

The objective was to evaluate the effects of dietary macronutrients and feeding frequency on blood glucose, insulin, total ghrelin and leptin. A total of twelve adult lean neutered male cats were used in three tests, all cross-over studies composed of a 15 d adaptation and blood sampling on day 16. In trial 1, differences between two- and four-meal feeding were tested. On day 16, blood samples were collected every 2 h for 24 h. In trial 2, macronutrient boluses were tested. Instead of the control diet, the morning meal on day 16 was replaced with an isoenergetic bolus of carbohydrate (maltodextrin), protein (chicken meat), fat or water. Fasted and ten postprandial blood samples were collected. In trial 3, diets high in fat (HF), protein (HP), carbohydrate (HC) or a control diet were tested. On day 16, fasted and ten postprandial blood samples were collected. Data were analysed to identify baseline and AUC changes. Cats fed four meals daily had greater (*P* = 0·03) leptin incremental AUC_0–24 h_ compared with cats fed twice daily. The carbohydrate bolus increased glucose (*P* < 0·001) and insulin (*P* < 0·001) incremental AUC_0–6 h_ and tended to increase (*P* = 0·09) leptin net AUC_0–6 h_. Cats fed the control and HC diets had greater (*P* = 0·03) glucose incremental AUC compared with the HF and HP conditions. Circulating hormone data were highly variable and indicated changes due to dietary macronutrients and feeding frequency, but further study is needed to identify impacts on appetite and contributing mechanisms.

In man and companion animals, obesity is one of the most common diseases and is a key risk factor for many other diseases. As in man, the incidence of obesity and type 2 diabetes mellitus in domestic cats has rapidly increased in recent decades^(^^1^^)^. In addition to the sedentary indoor lifestyle, the prevalence of highly palatable commercial pet foods (for example, high-fat dry diets) and/or inappropriate feeding strategies (for example, the amount and frequency of food provision) contribute to obesity^(^^2^^)^, insulin resistance^(^^3^^)^ and diabetes^(^^4^^)^ in domestic cats.

Diets containing different macronutrient concentrations may influence the release and circulating concentrations of appetite-regulating hormones, which could affect sensations of hunger, satiety and ultimately energy intake^(^^5^^–^^7^^)^. Ghrelin and leptin play competing roles in appetite regulation^(^^8^^)^ and the release of both has been reported to be affected by dietary nutrient composition^(^^9^^–^^13^^)^. Ghrelin, an orexigenic gastric hormone, stimulates food intake and supports lipogenesis^(^^14^^,^^15^^)^. In rodents and normal-weight human subjects consuming isoenergetic meals, ghrelin release is suppressed following a meal, but is macronutrient-specific^(^^11^^,^^16^^,^^17^^)^. Notably, fat appears to have a relatively weak ghrelin-suppressing capacity compared with carbohydrate and protein^(^^11^^,^^16^^,^^17^^)^. In contrast, leptin, mainly produced from adipose tissue, is an indicator of body energy status. It contributes to the long- and short-term regulation of food intake, acting on the hypothalamus to reduce appetite^(^^18^^,^^19^^)^ in rodents and humans. In human subjects, postprandial leptin concentrations have been reported to be dependent on dietary macronutrient composition; high-carbohydrate, low-fat meals result in higher postprandial leptin concentrations compared with high-fat, low-carbohydrate meals^(^^20^^,^^21^^)^. Very little is known regarding the effects of dietary macronutrients on the ghrelin and leptin response in cats^(^^22^^)^. The cat, as a true carnivore, relies on high-protein animal tissue to meet its specific nutritional requirements in the wild and is metabolically adapted to a lower glucose utilisation and higher protein metabolism when compared with most omnivores^(^^23^^)^. The unique metabolic need of cats underscores the importance of conducting this fundamental study in this species to increase our understanding and develop more specialised dietary strategies for weight management in cats.

In addition to diet composition, increased feeding frequency has been suggested to manage body weight (BW). To manage weight loss in cats, owners are often instructed to offer the daily food ration in several meals (between two and four) throughout the day rather than in a single meal^(^^24^^)^. Although feeding frequency has been studied for its potential impact on physical activity, recent studies have been more focused on the other side of the energy balance equation, namely, appetite control and food intake and how they may be affected by meal frequency^(^^25^^)^. Feeding frequency may have an impact on appetite control by influencing the release of appetite-regulating hormones, including insulin, ghrelin and leptin^(^^25^^–^^28^^)^.

Three tests were conducted in healthy adult cats to investigate how appetite-regulating hormone concentrations fluctuated over a 24 h period and responded to dietary manipulation. Our objectives were: (1) to monitor patterns of glucose, insulin, ghrelin and leptin concentrations over a 24 h period in cats fed a dry diet two or four times daily; (2) to measure the acute response of a single protein, fat or carbohydrate dose on postprandial glucose, insulin, ghrelin and leptin concentrations; and (3) to measure the effects of a protein-, fat- or carbohydrate-rich dry diet on fasting and postprandial glucose, insulin, ghrelin and leptin concentrations.

## Materials and methods

### Animals and diets

A total of twelve healthy adult, neutered, male domestic shorthair cats (initially aged 3 years; 4·74 (sem 0·16) kg BW; about five on a nine-point body condition score scale) were used in these three tests. Cats were group-housed in the same room (26·67 m^2^ × 3 m) for most of the day but were individually housed in cages (0·61 × 0·61 × 0·61 m) to access diets in the animal facility of the Edward R. Madigan Laboratory at the University of Illinois (Urbana, IL, USA). The room was environmentally controlled (20°C) with a 16 h light–8 h dark cycle. The 16 h light–8 h dark cycle has been used in our cat facility for many years due to practical reasons. It allows us to perform a variety of study designs and allows the collection of samples (blood, faecal or urine), feeding, weighing, etc. early in the morning or into the evening when the lights are on in the facility (06.00 to 22.00 hours). This photoperiod is similar to what occurs during the summer, so it is not an extreme photoperiod and should not have affected our data. Cats were provided access to various toys and scratching poles and socialised with each other and humans for behavioural enrichment.

Dietary ingredients and chemical composition of the four test diets are presented in Table 1. Test diets were extruded dry kibble diets based on milled brewer's rice, poultry by-product meal, maize gluten meal, whole yellow maize, whole wheat, soya protein isolate and fish meal. Test diets included: (1) the control diet (33 % metabolisable energy (ME) from each macronutrient); (2) the high-fat diet (HF diet: about 50 % ME from fat); (3) the high-protein diet (HP diet: about 50 % ME from protein); and (4) the high-carbohydrate diet (HC diet: about 50 % ME from carbohydrate). All diets were formulated to contain similar concentrations and type of dietary fibre so that any changes were due to macronutrient differences. Diets were formulated to meet all nutrient recommendations provided by the Association of American Feed Control Officials^(^^29^^)^ and were manufactured at the Nestlé Purina PetCare Product Technology Center. Before the nutritional trials, food intake was determined by calculating the daily maintenance energy requirement of lean domestic cats (ME requirement (kcal) = 100 × BW (kg)^0·67^; ME requirement (kJ) = 418 × BW (kg)^0·67^)^(^^30^^)^ and by using previous feeding records. Cats were weighed weekly and food intake was adjusted to maintain BW and body condition score throughout the study. If not consumed, food was removed, weighed and recorded. Water was available *ad libitum* throughout all trials.

**Table 1.** Ingredient and chemical composition of the test diets fed to cats

	Diet			
Item	Control	HF	HP	HC
Ingredient (%, as-fed basis)				
Brewer's rice	14·76	10·07	7·74	48·73
Chicken, whole carcass and part	19·21	16·84	20·14	19·52
Poultry byproduct meal	12·43	10·29	17·02	6·00
Maize gluten meal, 60 %	23·02	20·61	25·46	2·04
Whole yellow maize	6·88	6·47	–	–
Wheat flour	12·54	11·64	13·15	4·71
Soya protein isolate	–	–	10·04	8·84
Fish meal	2·21	1·29	1·55	1·50
Edible tallow with vitamin E	7·00	18·50	2·50	5·50
l-Lysine	0·04	0·03	0·46	0·45
Taurine	0·09	0·08	0·10	0·37
dl-Methionine	–	–	–	0·37
Calcium carbonate	0·07	0·58	–	0·07
Phosphoric acid	0·63	0·56	0·67	0·64
Potassium chloride	0·55	0·79	0·62	0·69
Salt	0·07	0·17	0·08	0·07
Pea fibre	–	1·56	–	–
Vitamin E, 50 %	0·02	0·02	0·02	0·02
Choline chloride, liquid	0·21	0·29	0·21	0·21
Mineral premix	0·18	0·16	0·19	0·19
Vitamin premix	0·06	0·05	0·06	0·06
Chemical composition (DM basis)				
DM (%)	94·30	94·22	93·41	92·56
Protein (%)	38·00	32·51	53·50	29·11
Acid-hydrolysed fat (%)	16·51	27·71	11·32	12·60
Total dietary fibre (%)	3·66	5·16	3·20	2·53
Ash (%)	8·12	6·89	6·90	5·66
Gross energy (kJ/g)	21·9	23·8	22·4	21·0
Gross energy (kcal/g)	5·23	5·68	5·35	5·01
ME (kJ/100 g DM)*	1637·28	1867·65	1553·31	1606·74
ME (kcal/100 g DM)*	391·32	446·38	371·25	384·02
Nutrients on energy basis (g DM/4184 kJ ME)				
Protein	97·11	72·83	144·11	75·80
Acid-hydrolysed fat	42·19	62·08	30·49	32·81
Total dietary fibre	9·35	11·56	8·62	6·59
NFE†	86·14	62·12	67·56	130·23
Macronutrients on energy basis (% ME)				
Protein	34·0	25·5	50·4	26·5
Acid-hydrolysed fat	35·8	52·8	25·9	27·9
NFE	30·2	21·7	23·7	45·6

HF, high fat; HP, high protein; HC, high carbohydrate; ME, metabolisable energy; NFE, N-free extract.

*ME was calculated using modified Atwater values with the assumptions that protein, fat and carbohydrate (NFE) provide 14·6, 35·6 and 14·6 kJ (3·5, 8·5, and 3·5 kcal) ME/g diet, respectively^(^^29^^)^.

† NFE (DM basis) was calculated using the following equation: (100 – crude protein – acid-hydrolysed fat – total dietary fibre – ash).

Before trial 1, body composition was determined using dual-energy X-ray absorptiometry (DXA). Cats were sedated and anaesthetised (intramuscular injection of a cocktail of butorphanol (0·2 mg/kg), medetomadine (0·02 mg/kg) and atropine (0·04 mg/kg)) and placed in ventral recumbency on the bed of a scanner (Hologic model QDR-4500 Fan Beam X-ray Bone Densitometer; Hologic Inc.) and with the use of computer software (Hologic Inc.) for cats, DXA data were used to determine body fat, lean and bone mineral content. All procedures were approved by the University of Illinois Institutional Animal Care and Use Committee before the studies.

### Experimental design

#### Trial 1

Because very little is known regarding appetite-regulating hormone concentrations in feline plasma, the initial test was designed to monitor daily fluctuations of glucose, insulin, total ghrelin and leptin concentrations in cats fed two or four meals per d. A total of twelve healthy adult male cats were used in a cross-over study design consisting of 32 d (two 16 d periods). In the first period, cats (six animals per treatment) were fed either two meals or four meals daily and vice versa for the second period. The control diet was fed in this trial. Half of the daily intake was offered to two-meal-fed cats at 08.00 and 20.00 hours. At 08.00, 12.00, 16.00 and 20.00 hours, one-quarter of the daily intake was offered to four-meal-fed cats, respectively. Cats were individually housed for access to diet from 08.00–09.00, 12.00–13.00, 16.00–17.00 and 20.00–21.00 hours each day. In each period, a 15 d adaptation phase was followed by a blood-sampling phase on day 16. A small blood sample (1·5 ml) was collected before the 08.00 hours meal (baseline samples) and then every 2 h for 24 h via a catheter.

#### Trial 2

This trial was designed to measure the acute response of a single protein, fat or carbohydrate bolus on postprandial glucose, insulin, total ghrelin and leptin concentrations. A total of twelve healthy adult male cats were used in a replicated 4 × 4 Latin square design for a total of 64 d (four 16 d periods). Cats were fed the control diet twice daily at 08.00 and 20.00 hours. A 15 d adaptation phase preceded a blood-collection phase on day 16. On day 16, rather than consuming the control diet, cats were dosed at 08.00 hours with one of the four treatments. Treatments included a 20 g carbohydrate load (20 g maltodextrin in about 20 ml water; about 335 kJ (80 kcal)), a 9 g fat load (lard; about 335 kJ (80 kcal)), a 27 g protein load (canned chicken; about 335 kJ (80 kcal); Sweet Sue Premium Chicken Breast) and 20 ml water. Water was used as a control for the effect of stomach filling, which has been shown not to influence postprandial ghrelin response in human subjects^(^^31^^)^. Carbohydrate and water solutions (about 20 ml) were given by slowly dripping the solution from the syringe into the mouth of the cats to accurately measure intake. Because of their high palatability, cats were able to consume the fat and protein loads without assistance. If time became an issue, cats were hand fed to increase consumption. Cats consumed all of the water, carbohydrate, fat and protein loads within 15 min. Before trial 2, cats were accustomed to these dosing strategies to minimise stress during testing, which could have affected the outcomes measured. Blood samples were collected before dosing (0 min) and at 15, 30, 60, 90, 120, 150, 180, 240, 300 and 360 min after dosing.

#### Trial 3

In this trial, fasting and postprandial responses to the HF, HP or HC diets were evaluated. A total of twelve healthy adult male cats were used in a replicated 4 × 4 Latin square design for a total of 64 d (four 16 d periods). Cats were randomly allocated to one of the four test diets listed in Table 1. Cats were fed twice daily at 08.00 and 20.00 hours and consumed their food within 10 min. After a 15 d adaptation phase, blood samples were collected on day 16 via a jugular or saphenous catheter before (0 min) and at 10, 20, 30, 60, 90, 120, 150, 180, 240, 300, 360 and 720 min after the morning meal was consumed.

### Chemical analyses

Diet subsamples were collected and ground using a Wiley mill (model 4; Thomas Scientific) through a 2-mm screen and dry ice in preparation for chemical analyses. Diet samples were analysed for DM and organic matter according to the AOAC (Association of Official Analytical Chemists)^(^^32^^)^. Crude protein was measured using a LecoNitrogen/Protein Determinator (model FP-2000; Leco Corporation) according to the AOAC^(^^32^^)^. Fat concentrations were determined by acid hydrolysis according to the AACC (American Association of Cereal Chemists)^(^^33^^)^ followed by ether extraction^(^^34^^)^. Total dietary fibre was determined according to Prosky *et al*.^(^^35^^)^. Gross energy was measured using a bomb calorimeter (model 1261; Parr Instrument Co.).

### Blood collection and analysis

The same blood collection and handling procedures for the measurement of blood glucose, plasma insulin and plasma total ghrelin and plasma leptin were used in all three tests. For all trials, jugular or saphenous catheters were placed 1 or 2 d before the collections to minimise stress. Cats were sedated and anaesthetised while performing the catheter placement by intramuscular injection of a cocktail of butorphanol (0·2 mg/kg), medetomadine (0·02 mg/kg) and atropine (0·04 mg/kg) along with the reversal atipamezole (0·02 mg/kg). Patency was maintained by flushing with heparinised saline daily until sampling began and following every sample. A total of 1·5 ml of blood was collected at each time point, maintaining the total volume of blood collection below the maximum recommended levels for the wellbeing of the cats. Catheters were removed after the last time point on the collection days.

Blood glucose concentration was immediately measured using the handheld AlphaTRAK blood glucose meter (Abbott Laboratories). Blood was then immediately transferred into a precooled Vacutainer tube (no. 367835; Becton, Dickinson and Company) containing EDTA and centrifuged at 1000 ***g*** at 4°C for 10 min. After centrifugation, plasma was collected into its respective cryovial and stored at –80°C until further analysis.

Before analysis, the kits were validated for use in our laboratory using parallel determination from increasing linear dilutions of pooled feline plasma (at least five cats) (data not reported). Plasma insulin was determined using the Feline Insulin ELISA kit (Mercodia) previously used in cats^(^^36^^)^. Following a 10-fold dilution, plasma total ghrelin concentration was analysed using the Total Ghrelin Canine ELISA kit (Phoenix Pharmaceuticals, Inc.). Plasma leptin concentration was measured using the Multi-species Leptin RIA kit (Millipore). The ghrelin and leptin kits have been used in cats in our laboratory previously^(^^37^^)^.

### Statistical analyses

For the baseline data (fasting samples), data were analysed using the MIXED procedure of SAS 9.2 (SAS Institute Inc.) testing the main effect (feeding frequency or test diet) and including random effects of cat and period. For postprandial data, incremental change from baseline (baseline subtracted) data were analysed to minimise any differences in baseline concentrations among cats and then analysed using the MIXED procedure of SAS 9.2 as repeated measures. The main effects of treatment and time were tested and the treatment × time interaction was evaluated if significant. Random effects of cat and period were included in the model. Means were separated for diets using the PDIFF statement in the MIXED procedure for individual time points after detecting a significant treatment effect using SLICE/time. AUC, as well as incremental AUC (IAUC), decremental AUC (DAUC) and net AUC (NAUC) data, were calculated using GraphPad Prism version 5.00 for Windows (GraphPad Software). Differences in the AUC of glucose, insulin, total ghrelin and leptin among treatments were tested for significance using the MIXED procedure of SAS 9.2. A probability of *P* ≤ 0·05 was considered significant and *P* ≤ 0·10 was considered a trend.

## Results

### Trial 1

**Fig. 1.**
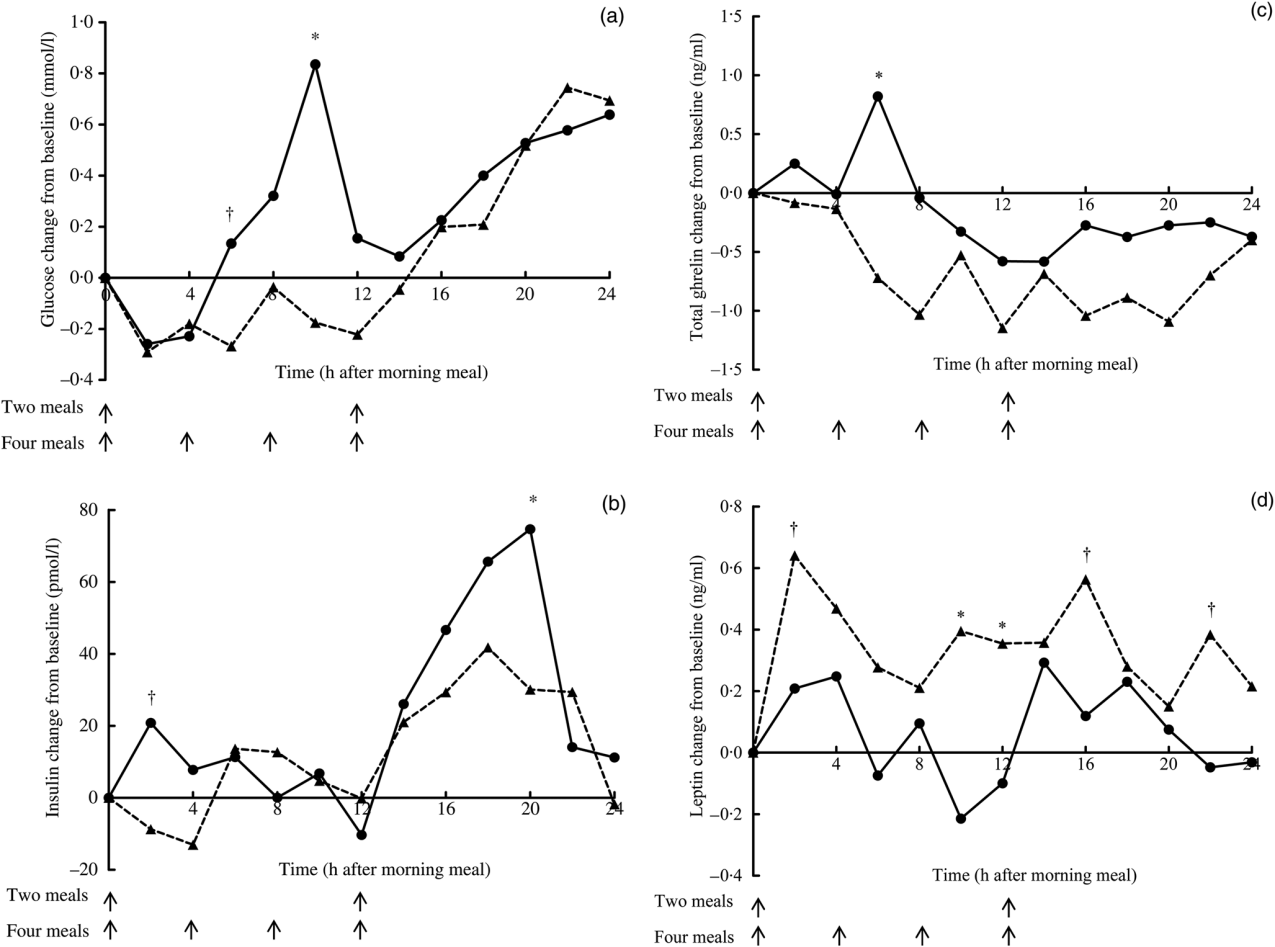
Mean incremental changes from baseline of blood glucose (a), insulin (b), total ghrelin (c) and leptin (d) in cats fed two meals (•) or four meals (▴) daily in trial 1. (a) Pooled sem = 0·19, treatment *P* = 0·008, time *P* < 0·0001, treatment × time *P* = 0·05. (b) Pooled sem = 13·99, treatment *P* = 0·06, time *P* < 0·0001, treatment × time *P* = 0·27. (c) Pooled sem = 1·6, treatment *P* = 0·93, time *P* = 0·38, treatment × time *P* = 0·91. (d) Pooled sem = 0·4, treatment *P* = 0·0001, time *P* = 0·28, treatment × time *P* = 0·69. *Mean values at a time point were significantly different (*P* ≤ 0·05). † Mean values at a time point were marginally significantly different (*P* ≤ 0·10).

Average food intake for all cats in this trial was 65·6 g/d (1074·0 kJ (256·7 kcal) ME/d) and was not different (*P* > 0·10) between periods. In cats fed two or four meals daily, the baseline concentrations of blood glucose (4·63 *v.* 4·46 mmol/l; sem 0·13 mmol/l), insulin (81·5 *v.* 67·1 pmol/l; sem 18·4 pmol/l), total ghrelin (7·2 *v.* 7·2 ng/ml; sem 1·0 ng/ml) and leptin (5·7 *v.* 5·4 ng/ml; sem 0·2 ng/ml) were not different. Fig. 1 presents incremental changes in blood glucose, insulin, total ghrelin and leptin concentrations over a 24 h period. Blood glucose concentrations of cats fed two meals daily were more variable than cats fed four meals daily during the light period. Cats fed two meals daily had two peaks of glucose concentration in both the light and dark periods. Similar to glucose, insulin concentrations of cats fed two meals daily were also more variable and maintained a higher concentration throughout the 24 h period compared with those fed four meals daily. Total ghrelin remained below baseline throughout the 24 h period in cats fed four meals daily, but its concentrations remained above baseline during the light period from 08.00 to 16.00 hours in cats fed two meals daily. Cats fed four meals daily maintained higher leptin concentrations over the 24 h period than cats fed two meals daily. Cats fed four meals daily had greater (*P* = 0·03) leptin IAUC_0–24 h_ compared with cats fed twice daily (10·8 *v.* 5·5 ng/ml, sem 2·1 ng/ml). However, AUC_0–24 h_ of glucose, insulin and total ghrelin were not affected by feeding frequency.

### Trial 2

**Fig. 2.**
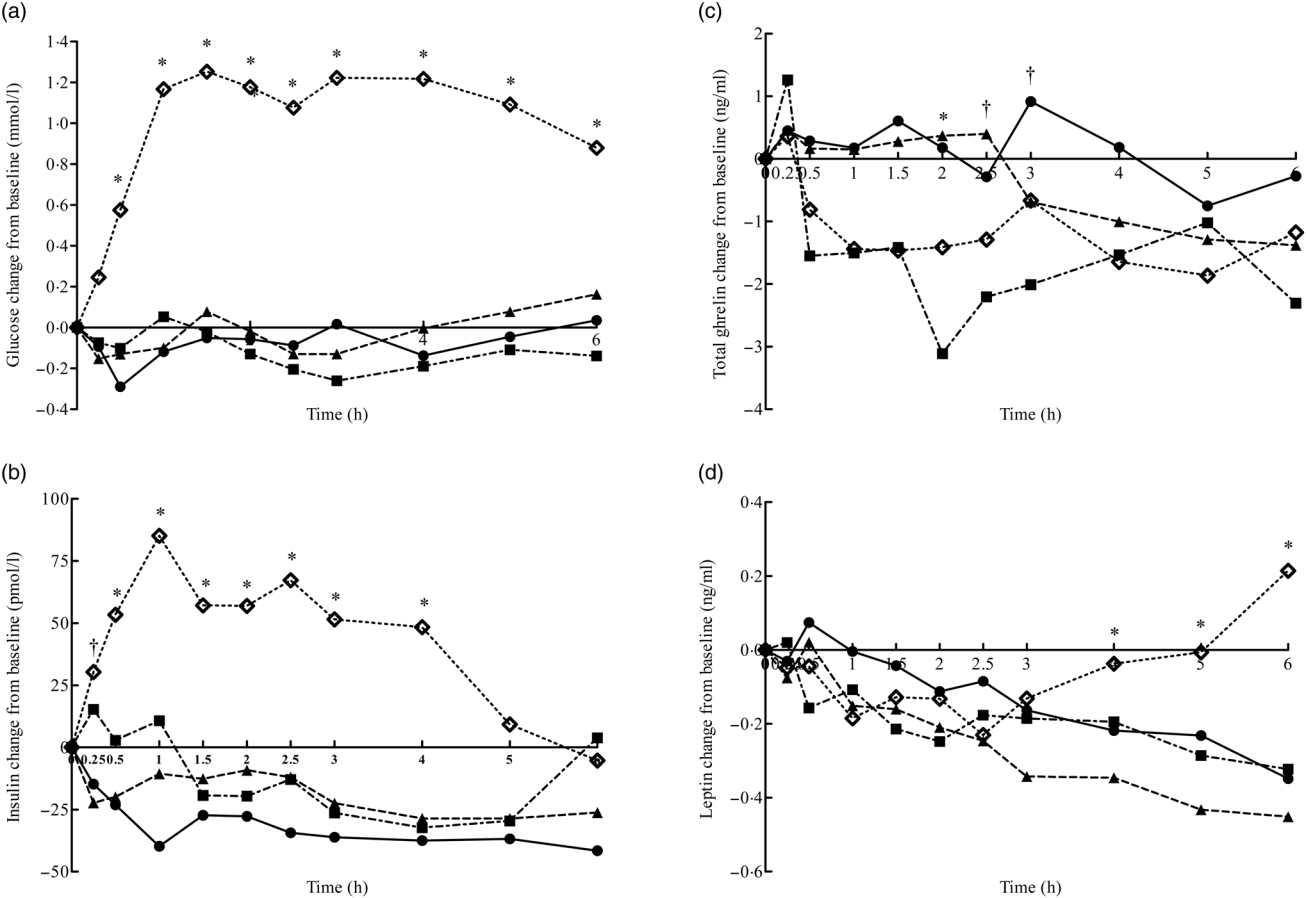
Mean incremental changes from baseline of blood glucose (a), insulin (b), total ghrelin (c) and leptin (d) in cats fed water (●), lard (fat; ■), canned chicken (protein; ▴) and maltodextrin (carbohydrate; ◊) in trial 2. (a) Pooled sem = 0·19, treatment *P* < 0·0001, time *P* = 0·02, treatment × time *P* = 0·0002. (b) Pooled sem = 17·1, treatment *P* < 0·0001, time *P* = 0·08, treatment × time *P* = 0·06. (c) Pooled sem = 0·9, treatment *P* < 0·0001, time *P* = 0·04, treatment × time *P* < 0·0001. (d) Pooled sem = 0·1, treatment *P* < 0·0001, time *P* < 0·0001, treatment × time *P* = 0·0002. * Mean values at a time point were significantly different (*P* ≤ 0·05). † Mean values at a time point were marginally significantly different (*P* ≤ 0·10).

Average food intake for all cats in this trial was 56·9 g/d (931·8 kJ (222·7 kcal) ME/d) and was not different (*P* > 0·10) among periods. Baseline glucose, insulin, total ghrelin and leptin concentrations of cats dosed with water, fat, protein or carbohydrate were not different (Table 2). Fig. 2 presents incremental blood glucose, insulin, total ghrelin and leptin concentrations over a 6 h postprandial period. The carbohydrate load produced a marked increase in incremental glucose concentration after 30 min and reached a plateau at 1 h. Glucose concentration remained elevated over the 6 h and was greater (*P* = 0·0002) than for the water, fat and protein conditions overall, resulting in greater (*P* < 0·001) IAUC_0–6 h_ than cats dosed with water, fat or protein. Similar to glucose, incremental insulin concentrations of cats dosed with carbohydrate rapidly increased and reached their peak at 1 h and remained higher than for the other treatments for 4 h postprandially (Fig. 2(b)). Cats dosed with carbohydrate had greater (*P* < 0·001) insulin IAUC_0–6 h_ compared with cats fed the other three treatments (Table 2).

**Table 2.** Blood glucose, insulin, total ghrelin and leptin concentrations in cats dosed with water, lard (fat), canned chicken (protein) or maltodextrin (carbohydrate) (trial 2) (Mean values and pooled standard errors; *n* 12)

Item	Water	Fat	Protein	Carbohydrate	Pooled sem	*P*
Baseline concentration						
Glucose (mmol/l)	4·55	4·60	4·53	4·44	0·17	0·78
Insulin (pmol/l)	78·4	74·6	75·2	70·2	15·8	0·98
Ghrelin (ng/ml)	8·5	9·9	9·2	9·3	1·1	0·45
Leptin (ng/ml)	3·1	3·1	3·1	3·1	0·3	0·99
IAUC_0–6 h_						
Glucose (mmol/l × h)	0·75^a^	0·58^a^	0·94^a^	6·34^b^	0·56	<0·001
Insulin (pmol/l × h)	24·1^a^	61·2^a^	31·6^a^	300·7^b^	39·7	<0·001
Ghrelin (ng/ml × h)	3·9	2·7	2·5	1·0	1·5	0·60
Leptin (ng/ml × h)	0·4	0·1	0·1	0·4	0·2	0·39
DAUC_0–6 h_						
Glucose (mmol/l × h)	1·28^a^	1·37^a^	1·06^a,b^	0·06^b^	0·46	0·08
Insulin (pmol/l × h)	204·5^a^	153·1^a,b^	131·3^a,b^	44·7^b^	49·6	0·10
Ghrelin (ng/ml × h)	3·1	12·3	5·4	8·6	3·4	0·19
Leptin (ng/ml × h)	1·2	1·3	1·8	0·8	0·4	0·14
NAUC_0–6 h_						
Glucose (mmol/l × h)	−0·53^a^	−0·79^a^	−0·09^a^	6·29^b^	0·85	<0·001
Insulin (pmol/l × h)	−199·7^a^	−92·6^a^	−101·0^a^	256·3^b^	72·1	<0·001
Ghrelin (ng/ml × h)	0·8	−9·6	−2·9	−7·7	4·0	0·19
Leptin (ng/ml × h)	−0·9^x,y^	−1·2^x,y^	−1·7^x^	−0·4^y^	0·5	0·09

IAUC, incremental AUC; DAUC, decremental AUC; NAUC, net AUC.

^a,b,c^ Mean values within a row with unlike superscript letters were significantly different (*P* ≤ 0·05).

^x,y,z^ Mean values within a row with unlike superscript letters were marginally significantly different (*P* ≤ 0·10).

Incremental total ghrelin concentrations varied greatly in response to carbohydrate, fat and protein and tended to stay below baseline values (Fig. 2(c)). Although a treatment × time interaction (*P* < 0·0001) was significant, postprandial IAUC_0–6 h_, DAUC_0–6 h_ and NAUC_0–6 h_ of total ghrelin were not affected by the treatments (Table 2). Total ghrelin concentrations remained close to baseline in cats dosed with water.

Incremental leptin concentrations decreased below baseline for all treatments. Although leptin continued to decrease in cats dosed with water, protein and fat, incremental leptin concentrations in cats dosed with carbohydrate started increasing at 2·5 h and reached baseline at 5 h (Fig. 2(d)). When compared with cats dosed with carbohydrate, those dosed with protein tended to have a decreased (*P* = 0·09) leptin NAUC_0–6 h_ than with the carbohydrate load.

### Trial 3

Average food intake for cats fed the control, HF, HP and HC diets in this trial was 51·5 g/d (843·1 kJ (201·5 kcal) ME/d), 46·2 g/d (862·3 kJ (206·1 kcal) ME/d), 54·4 g/d (845·2 kJ (202·0 kcal) ME/d) and 54·3 g/d (871·5 kJ (208·3 kcal) ME/d), respectively, which was not different (*P* > 0·10) among diets. Diets were highly palatable for all cats (no food refusals) and no digestibility issues were observed throughout the entire trial.

Baseline glucose, insulin, total ghrelin and leptin concentrations did not differ among the dietary treatments (Table 3). Incremental blood glucose concentration in cats fed the control diet was greater than for the other diets between 1·5 and 3 h postprandially, while cats fed the HC diet maintained a greater incremental glucose concentration after 5 h postprandially (data not shown). The glucose IAUC_0–6 h_ was higher (*P* = 0·03) in cats fed the control diet compared with those fed the HF and HP diets and the glucose IAUC_0–12 h_ was higher (*P* = 0·03) in cats fed the HC diet compared with the HF and HP diets. The IAUC, DAUC and NAUC of insulin, total ghrelin and leptin were not affected by diets over 6 or 12 h postprandially (Table 3).

**Table 3.** Blood glucose, insulin, total ghrelin, and leptin concentrations in cats fed the control, high-fat (HF), high-protein (HP) and high-carbohydrate (HC) diets (trial 3) (Mean values and pooled standard errors; *n* 12)

	Diet					
Item	Control	HF	HP	HC	Pooled sem	*P*
Baseline concentration						
Glucose (mmol/l)	4·93	4·92	4·86	4·98	0·26	0·91
Insulin (pmol/l)	84·3	65·5	71·7	86·8	23·4	0·58
Ghrelin (ng/ml)	6·7	6·4	6·1	6·7	0·6	0·52
Leptin (ng/ml)	4·3	4·1	4·0	4·3	0·3	0·40
IAUC_0–6 h_						
Glucose (mmol/l × h)	3·32^a^	1·62^b^	1·66^b^	2·79^a,b^	0·57	0·03
Insulin (pmol/l × h)	174·9	101·0	192·2	162·5	62·9	0·52
Ghrelin (ng/ml × h)	4·8	1·6	0·9	1·2	2·0	0·50
Leptin (ng/ml × h)	0·4	0·7	0·4	0·9	0·3	0·43
DAUC_0–6 h_						
Ghrelin (ng/ml × h)	2·9	3·7	4·1	3·1	1·2	0·87
Leptin (ng/ml × h)	1·1	1·4	1·7	1·3	0·4	0·65
NAUC_0–6 h_						
Ghrelin (ng/ml × h)	2·0	−2·2	−3·2	−1·9	2·6	0·48
Leptin (ng/ml × h)	−0·8	−0·7	−1·3	−0·4	0·5	0·60
IAUC_0–12 h_						
Glucose (mmol/l × h)	8·09^a,c^	4·66^b,c^	3·85^b^	8·27^a^	1·45	0·03
Insulin (pmol/l × h)	256·6	278·0	347·0	188·8	97·8	0·92
Ghrelin (ng/ml × h)	5·5	2·9	2·0	3·2	2·2	0·70
Leptin (ng/ml × h)	0·7	1·2	0·6	1·6	0·6	0·54
DAUC_0–12 h_						
Ghrelin (ng/ml × h)	6·8	7·1	9·4	6·0	2·9	0·83
Leptin (ng/ml × h)	2·7	3·9	3·4	3·4	0·9	0·82
NAUC_0–12 h_						
Ghrelin (ng/ml × h)	−1·2	−4·2	−7·4	−2·8	4·0	0·69
Leptin (ng/ml × h)	−2·0	−2·8	−2·8	−1·7	1·3	0·90

IAUC, incremental AUC; DAUC, decremental AUC; NAUC, net AUC.

^a,b,c^ Mean values within a row with unlike superscript letters were significantly different (*P* ≤ 0·05).

## Discussion

The aim of the present study was to investigate the potential role of dietary macronutrient composition on circulating appetite-regulating hormone concentrations in healthy cats. Three trials were conducted to: (1) observe the daily hormonal fluctuations in response to different meal patterns; (2) evaluate the postprandial response to oral ingestion of a single macronutrient; and (3) evaluate the fasting and postprandial responses to macronutrient-rich diets.

Because very little is known regarding appetite-regulating hormone concentrations in cats and their relationship with feeding frequency, the initial trial was designed to monitor the daily fluctuation of circulating glucose, insulin, total ghrelin and leptin concentrations in cats fed two or four meals per d. We hypothesised that increasing feeding frequency without changing daily food intake would prevent large metabolic and hormonal fluctuations. In that initial trial, circulating glucose and insulin concentrations were less variable in cats fed four compared with two meals daily, which is consistent with data in healthy and overweight human subjects^(^^25^^,^^38^^)^ and with feeding strategies recommended for diabetic cats. We also observed that cats fed four meals daily had lower incremental ghrelin (Fig. 2(c)) and greater incremental leptin (Fig. 2(d)) concentrations and leptin IAUC_0–24 h_ compared with cats fed two meals daily. These data indicate that increasing feeding frequency may inhibit ghrelin secretion and stimulate leptin secretion, which may aid in appetite control. Smeets & Westerterp-Plantenga^(^^39^^)^ reported that eating three compared with two meals increased fat oxidation and feelings of satiety over a 24 h period in healthy women. Leptin can activate the enzyme AMP kinase in peripheral tissues and is important in regulating lipid oxidation^(^^40^^)^. While greater leptin secretion with increased feeding frequency may contribute to decreased appetite and/or to increased fat oxidation, these outcomes were not tested in the present study. Limitations exist in our current test, with the primary one being the sampling times. Because the blood volume allowances over 24 h limited the number of samples we could collect, the changes occurring between every 2 h collection were unknown. Therefore, our perception of the hormonal responses could have been influenced by the selection of blood sampling times. More accurate hormonal responses could be accomplished by smaller sample volume requirements in future studies. Another potential limitation of the protocol used is that we used meal feeding, which may differ from *ad libitum* feeding. Although this may result in physiological and metabolic differences from some household cats that have free access to food, this method allowed concise and accurate food consumption and postprandial blood measurements.

Before evaluating complex diets, we designed a trial to measure the acute response of a single macronutrient dose. To our knowledge, no study has tested the effect of a single macronutrient or a macronutrient-rich diet on postprandial ghrelin and leptin concentrations in cats. We hypothesised that a carbohydrate load would have the most rapid and effective influence on postprandial glucose and insulin concentrations. We also hypothesised that the fat load would have a relatively weak effect on ghrelin suppression and leptin secretion, whereas protein, which is considered the most satiating macronutrient in humans, would have a prolonged effect on ghrelin suppression and leptin secretion. Macronutrient sources that were deemed to be highly digestible and contained relatively large amounts of one macronutrient were selected. The amount of each macronutrient dose fed to cats was based on the amount of energy provided by each macronutrient (about 335 kJ; 80 kcal) and how it compared with daily intake (approximately 25 % of daily ME). Based on a similar canine study performed in our laboratory^(^^27^^)^ and other human studies^(^^10^^,^^12^^,^^31^^)^, a 6 h length of blood sampling was selected because the postprandial ghrelin response to diet was expected to return to baseline by then.

Similar to the previous studies in dogs^(^^27^^)^ and cats^(^^41^^)^, we observed that the oral carbohydrate load elicited a rise in blood glucose and insulin and that their IAUC_0–6 h_ were higher than those when water, fat and protein were given. Due to these robust increases in postprandial glucose and insulin in the present study, significant ghrelin suppression after carbohydrate load was expected to be observed simultaneously. Although we did not observe the influence of macronutrient loads (fat, protein and carbohydrate) on ghrelin DAUC_0–6 h_, all macronutrient loads suppressed ghrelin secretion 6 h postprandially. Our observations that ghrelin was mostly responsive to carbohydrate and fat loads are consistent with previous findings in human subjects^(^^31^^,^^42^^)^. Blom *et al.*^(^^31^^)^ reported that postprandial ghrelin responded rapidly and dose-dependently to carbohydrate intake and might be regulated through insulin. Erdmann *et al.*^(^^42^^)^ reported that a fat-rich diet decreased plasma ghrelin levels, but reached a nadir later than when carbohydrates were fed. Protein load showed the weakest ghrelin response in cats compared with fat and carbohydrate loads. This finding is inconsistent with previous human studies in which protein induced a prolonged postprandial ghrelin suppression^(^^10^^,^^43^^–^^45^^)^. There are a few other studies in human subjects, however, that suggested that protein ingestion stimulated^(^^42^^)^ or had no effect on^(^^46^^)^ postprandial ghrelin concentration.

Factors other than macronutrient composition may affect ghrelin response. Arosio *et al.*^(^^47^^)^ reported that circulating ghrelin concentrations were decreased in human subjects as much by sham feeding as they were by meal consumption, suggesting the importance of the cephalic response to nutrient intake and the role of vagal activity in the control of ghrelin secretion. However, the role for cephalic–vagal stimulation on ghrelin suppression is unclear in cats. It might be argued that the volume difference that existed among our macronutrient loads influenced postprandial ghrelin secretion. This was probably not the case, however, because previous studies demonstrated that the gastric factor alone (such as stomach expansion) does not play a role in the regulation of ghrelin secretion^(^^48^^,^^49^^)^.

In contrast to our hypothesis that postprandial leptin secretion would increase, leptin actually decreased in the present study. It is unknown whether the decreased leptin secretion in the initial 2·5 h after dosing carbohydrate was due to the large amount of the highly digestible macronutrient selected or the dosing method used in the present study. A potential drawback of carbohydrate loads was the incidence of diarrhoea in the present study, which has also been reported in cats given an oral glucose tolerance test^(^^41^^)^. Dietary carbohydrate has been reported to cause gastrointestinal disturbances in cats due to its osmotic effect if the amount eaten exceeds the digestive capacity of the small intestine^(^^50^^)^. Although diarrhoea was only present on the day of the bolus, it may have contributed to the variability of the results. Our observation that leptin concentration in cats dosed with carbohydrate started increasing after 2·5 h postprandially may indicate the contribution of leptin on increasing the glucose uptake as well as the potential regulating effect of insulin on leptin. Postprandial leptin concentrations remained below baseline after fat ingestion in the present study, which is consistent with previous findings in lean and obese human subjects^(^^12^^,^^51^^)^. It has been suggested that the primary role of leptin in the regulation of energy homeostasis is a response to negative energy balance: leptin decreases during starvation, triggering an increased feeling of hunger^(^^52^^)^. Protein had a weaker effect on postprandial leptin secretion compared with carbohydrate, indicating a reduced satiating effect of protein in cats. It is correlated with the postprandial ghrelin response to protein in the present study.

To apply this research to a more practical scenario, responses to three isoenergetic dry kibble diets that had different macronutrient profiles were tested in the present study. From the control diet, which was based on a commercially available cat food, similar ingredients were used in different quantities to formulate a wide energy distribution in terms of macronutrient content (approximately 50 % of ME from each macronutrient). We expected to observe similar postprandial responses to macronutrients in trials 2 and 3. The diet containing a high carbohydrate content increased postprandial glucose in a similar manner, but did not lead to differences in baseline or postprandial insulin, ghrelin and leptin concentrations. Cats fed the control and HC diets had a similar increase in postprandial glucose, but failed to increase insulin secretion. Farrow *et al.*^(^^53^^)^ reported a similar result where a high-carbohydrate diet resulted in a greater postprandial glucose AUC when compared with high-protein and high-fat diets in healthy non-obese cats, while insulin AUC only tended to be increased in cats fed a high-carbohydrate diet. Coradini *et al*.^(^^22^^)^ reported that a high-carbohydrate, low-protein diet resulted in higher postprandial glucose and insulin concentrations compared with a low-carbohydrate, high-protein diet in cats fed to maintain BW. The feeding of high-carbohydrate diets has been suggested to increase the risk for developing diabetes in cats^(^^4^^)^. Hoenig *et al*.^(^^54^^)^ suggested that cats fed a high-carbohydrate, low-protein diet were more prone to develop obesity and insulin resistance compared with those fed a high-protein, low-carbohydrate with the same energy intake, mainly because high-protein diet led to greater heat production. In the present study, however, heat production was not measured and the HP diet did not lead to differences in glucose or insulin AUC.

In the present study, body fat percentage, as measured by DXA, was 14·1 % fat in the cats studied. Because we were first interested in assessing feeding frequency and macronutrient responses in healthy non-obese cats, it must be noted that the dietary effects reported here may not be consistent with those found in obese cats. For example, Hoenig *et al.*^(^^54^^)^ reported that obesity, but not dietary content, led to severe insulin resistance in cats and a marked decrease in glucose effectiveness, indicating that postprandial glucose and hormonal responses were affected more by body composition than dietary composition. Further research is needed to determine the effect of the macronutrient-rich test diets on appetite regulation in obese cats.

In conclusion, the present study presents novel data regarding the effects of feeding frequency and dietary macronutrient composition on postprandial glucose, insulin, total ghrelin and leptin concentrations in healthy non-obese adult cats. These data may provide a foundation for better understanding into the mechanisms of appetite regulation by dietary macronutrient manipulation. Even though circulating hormones were highly variable, our data suggested that dietary macronutrients affected postprandial insulin, total ghrelin and leptin secretions. Interestingly, dietary protein was observed to have a relatively weak effect on postprandial total ghrelin and leptin concentrations. Diets containing higher carbohydrate content increased blood glucose, but did not appear to affect appetite-regulating hormone concentrations in non-obese cats. Given the variability observed in the meal test study, increased numbers of animals will be required in future studies to identify the impact of macronutrients on appetite. Moreover, identifying the relationship between dietary macronutrients and appetite regulation in obese or diabetic cats may be more meaningful and may aid in the development of weight-loss or diabetic diets. Further research is also needed to compare these responses in *ad libitum*-fed *v.* meal-fed cats.
